# Feasibility of a blood biomarker cohort study within a memory clinic service

**DOI:** 10.1002/alz70856_100598

**Published:** 2025-12-25

**Authors:** Jian Xiang Ding, Oneil G Bhalala, Kai Sin Chin, Jenny Yu, Sarah Holper, Dina LoGiudice, Leonid Churilov, Rosie Watson, Nawaf Yassi

**Affiliations:** ^1^ The Walter and Eliza Hall Institute of Medical Research, Parkville, VIC, Australia; ^2^ Royal Melbourne Hospital, Parkville, VIC, Australia; ^3^ The University of Melbourne, Melbourne, VIC, Australia

## Abstract

**Background:**

Patient recruitment is a major challenge in dementia clinical studies, particularly within an aged population due to complex comorbidities, and within culturally and linguistically diverse (CALD) populations. We aim to describe the establishment and early recruitment experience in our ongoing blood biomarker cohort study based within a public hospital memory clinic service.

**Method:**

Patients who were undergoing diagnostic assessment in the Cognitive, Dementia, and Memory Service (CDAMS) clinic at the Royal Melbourne Hospital were recruited beginning in October 2023. Inclusion criteria were broad to include participants of any age where telephone/telehealth follow‐up was deemed feasible at 12 months. Patients requiring interpreters and/or medical treatment decision makers were not excluded. In addition to standard of care, participants provided a single blood sample within 6 months of recruitment and had a 12‐month telephone/telehealth study visit to confirm final diagnosis. Blood biomarkers including plasma neurofilament light chain and phospho‐tau‐217 will be investigated for their diagnostic performance in this cohort.

**Result:**

73 participants with a total recruitment target of 200 have been recruited with 90% of approached patients consenting to participate in the study. The mean age of participants is 78 years (SD 8.5 years) and 63% of participants are female. 21% of participants are from CALD populations.

**Conclusion:**

This study demonstrates the feasibility of dementia blood biomarker studies in clinical settings using pragmatic study designs and strategies to mitigate barriers to recruitment. By minimizing exclusion criteria and visit burden, such studies can recruit a diverse and clinically‐relevant population.

